# Supplementary figures and images for: Genome-Wide-Association-Analysis-Based Identification of Genetic Loci and Candidate Genes Associated with Cold Germination in Sweet Corn
Source: Biology (Basel). 2025 May 21;14(5):580. doi: 10.3390/biology14050580 (PMC12109514; doi:10.3390/biology14050580)

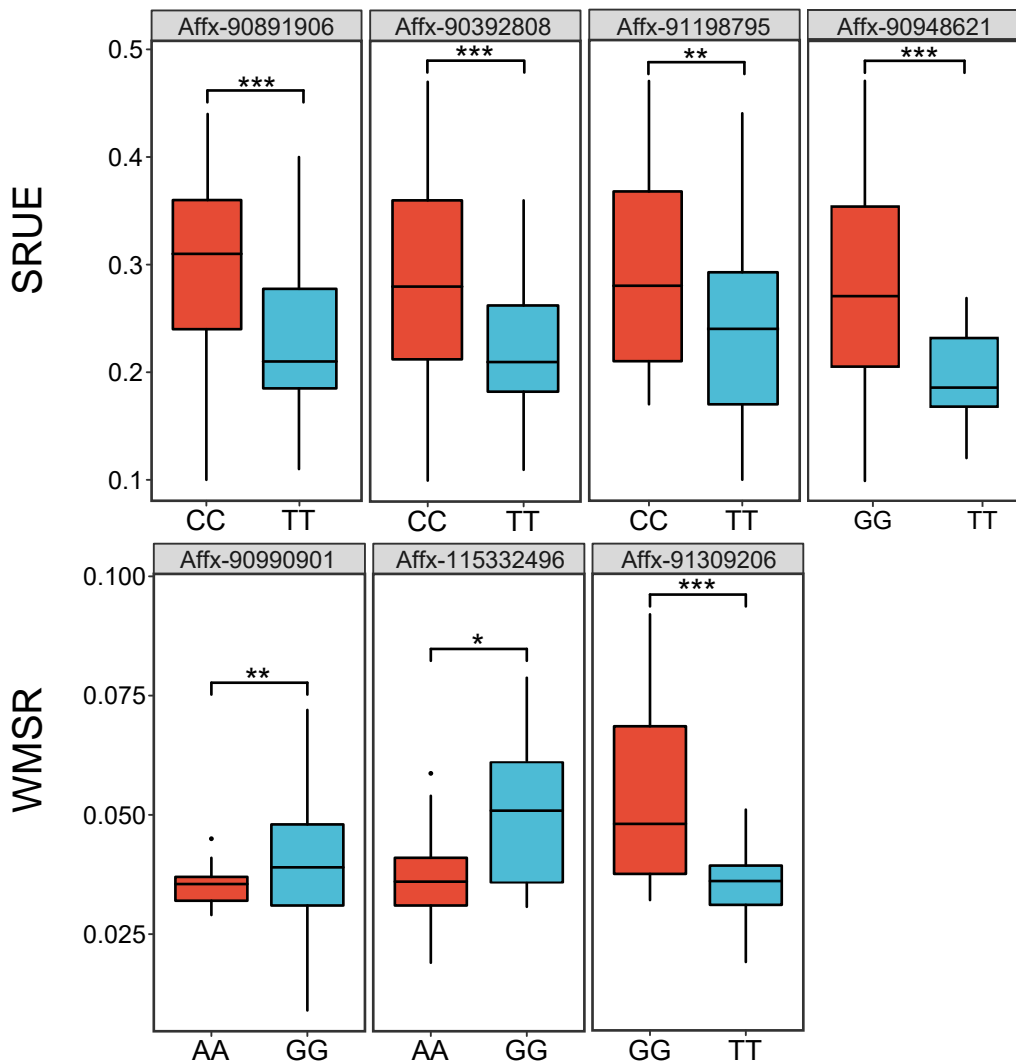

Supplement: Supplementary file 1 [file biology-14-00580-s001.zip › Supplementary Figure S1.pdf]
